# Special Issue “Potential Neuromodulatory Profile of Phytocompounds in Brain Disorders”

**DOI:** 10.3390/molecules22010040

**Published:** 2016-12-28

**Authors:** Luigia Trabace, Maria Grazia Morgese

**Affiliations:** Department of Clinical and Experimental Medicine, University of Foggia, Via Napoli, 20, Foggia 71121, Italy; mariagrazia.morgese@unifg.it

Several lines of evidence have highlighted that herbal preparations hold great potential for the treating of brain disorders, ranging from neurodegenerative to neuropsychiatric diseases. Phytocompounds have been shown to easily pass the blood brain barrier, thereby influencing the cerebral neurochemical and functional pathways. In vitro and in vivo studies have underlined a key role of medicinal plants in maintaining brain functioning through the modulation of the expression of different receptors, signal transduction pathways, transcription factors, and neurotransmitter release.

In this special issue, a team of international experts discusses all the most relevant topics in regard to the potential use of plant-derived chemicals as a potential and promising class of therapeutics for the treatment of psychiatric and neurodegenerative disorders.

In the search for novel substrates useful to obtain promising natural bioactive compounds, microalgae represent a novel field yet to be explored. In particular, the green microalga *Chlorella*, can be used as natural source to obtain a whole variety of compounds, such as omega (ω)-3 and ω-6 polyunsaturated fatty acids. Morgese and colleagues [1] report in an original work the memory- enhancing properties of a lipid extract of *Chlorella sorokiniana* in rats. This behavioural outcome was associated to a selective increase in serotonin and noradrenaline content in the hippocampal area, pointing towards a beneficial effect of *Chlorella sorokiniana* extract on short-term memory.

Novel complementary therapy for the treatment of neurological disorders, such as epilepsy, is another possible treatment approach in a context of a multitarget pharmacological strategy. In this regard, Citraro et al., [2] have shown that flavonoid-rich extract from orange juice displays anti-convulsant properties in murine models of epilepsy. Such an effect is likely mediated by inhibition of NMDA receptors at the glycine-binding site and by acting as an agonist on the benzodiazepine-binding site at GABA_A_ receptors.

Among neurological disorders, Alzheimer’s disease (AD) is a neurodegenerative disease that represents the most common form of dementia in elderly people. However, the treatment options are nowadays still very limited. Acetylcholinesterase (AChE) inhibitors remain the first choice of drugs for the treatment of AD. Various plant-derived compounds are already used for the treatment of AD and they represent a promising source of new bioactive compounds with anti-AChE activity. In this regard, Kaufmann and co-workers have evidenced in their research that traditional Chinese medicines, such as extracts of *Berberis bealei* (formerly *Mahonia bealei*), *Coptis chinensis* and *Phellodendron chinense*, very rich in isoquinoline alkaloids, inhibit AChE via synergistic interaction of their secondary metabolites. These drugs may represent an alternative and less expensive anti-AChE-based cure for AD [3]. The protective role of phytocompounds in neurodegeneration has also been covered in this special issue by the work of Cirmi et al., who reviewed the most prominent findings in the literature related to *Citrus*-derived flavonoids [4]. Interestingly, Sawamoto et al. found in their original research that 3,5,6,7,8,3′,4′-heptamethoxyflavone (HMF), a *Citrus* flavonoid, exerts antidepressant effects by inducing the expression of brain-derived neurotrophic factor (BDNF). In particular, HMF treatment was shown to ameliorate corticosterone-induced depression-like behavior, and corticosterone-induced reductions in BDNF production in the hippocampus. In addition, HMF treatment restored corticosterone-induced reductions in neurogenesis in the dentate gyrus subgranular zone and corticosterone-induced reductions in the expression levels of phosphorylated calcium-calmodulin-dependent protein kinase II and extracellular signal-regulated kinase1/2 [5]. Neuroprotective and antidepressant effects have also been reported for a chlorogenic acid (CGA)-enriched extract from *Eucommia ulmoides* in the article of Wu and co-workers. The authors showed for the first time in vivo that CGA can cross the blood-cerebrospinal fluid barrier, is neuroprotective and promotes serotonin release through enhancing synapsin I expression [6].

Furthermore, neurodegenerative disorders are very often accompanied by cognitive decline. Mazzanti and Di Giacomo show in their review article the state of art and they discuss the disappointing and inconclusive results originated by clinical trials investigating curcumin and resveratrol in the prevention or treatment of cognitive disorders. The authors conclude by encouraging long-term exposure clinical trials with well standardized preparations and with high bioavailability [7]. In addition, AD and other neurological disorders are often characterized by dementing processes. Dementia describes a class of heterogeneous diseases with still unravelled etiopathogenetic mechanisms. In this regard, Libro and colleagues have drawn in their manuscript an overview of literature evidences related to phytocompounds with demonstrated preventive properties for dementia [8].

On the other hand, oxidative stress-mediated cellular injury has been considered as a major cause of neurodegenerative diseases, including AD, thus antioxidant-based therapies may represent a great potential option to slow down neurodegenerative progression. Cheong et al. have reported that costunolide (CS), a known sesquiterpene lactone, is an useful scavenger of reactive oxygen species, stabilizes the mitochondria membrane potential, and is able to reduce apoptosis-related protein, such as caspase 3, as well as to inhibit of phosphorylation of p38 and the extracellular signal-regulated kinase [9].

Ultimately, in this special issue neuronal impairment secondary to chemical exposure, particularly to chemotherapy drugs, it has also been taken into account. In this regard, Lee and Kim revised literature data available concerning phytochemicals and medicinal herbs on chemotherapy-induced peripheral neuropathy, a frequent adverse effect of neurotoxic anticancer medicines [10]. In this light, Kim et al. reported in their original research a potent anti-allodynic effect of Cinnamomi Cortex, a widely used medicinal herb in East Asia for cold-related diseases, in oxaliplatin-injected rats through inhibiting spinal glial cells and pro-inflammatory cytokines [11].

In conclusion, we hope that this special issue will result intriguing for the readers and will prompt to further research in this novel field. We take the occasion to thank all the contributors for the great work carried out.
